# Dietary salt concentrations influence growth, nutrient utilization, and fatty acid profiles of turbot (*Scophthalmus maximus*) reared in brackish water

**DOI:** 10.1007/s10695-024-01391-w

**Published:** 2024-08-10

**Authors:** Hüseyin Sevgili, Adem Kurtoğlu, Masahiro Oikawa, Faruk Pak, Özgür Aktaş, Firdevs Mert Sivri, O. Tufan Eroldoğan

**Affiliations:** 1 Fisheries Application and Research Center & Department of Aquaculture, Eğirdir Fisheries Faculty, Isparta University of Applied Sciences, Eastern Campus, 32260 Isparta, Turkey; 2 Mediterranean Fisheries Research Production and Training Institute, Beymelek Unit, Demre, Antalya, Turkey; 3 Department of Basic Pharmaceutical Sciences, Faculty of Pharmacy, Süleyman Demirel University, Isparta, 32200 Turkey; 4 Department of Aquaculture, Faculty of Fisheries, Çukurova University, 01330 Balcalı, Adana, Turkey

**Keywords:** Turbot, Dietary salt, Growth, Tissue fatty acid composition

## Abstract

Expansion of economically viable turbot (*Scophthalmus maximus*) aquaculture depends on access to brackish-cold ground water sources in various parts of the world. Since brackish water sources can adversely affect the physiology and zoo technical performance of fish due to the burden of osmoregulation, dietary salt inclusion can alleviate the negative impacts of low-saline waters in several aquaculture species. This study investigated the effects of increasing dietary salt levels on the growth, feed utilization, body composition, and tissue fatty acid composition of juvenile turbot (initial live weight 120.3 ± 0.03 g/fish). Fish were fed five experimental diets supplemented with varying levels of sodium chloride (1.8–6.4%) or a control diet without salt. Each diet was tested in triplicate tanks for 9 weeks. Results showed that increasing dietary salt intake negatively impacted turbot performance, with significant reductions in weight gain, specific growth rate, and feed conversion ratio. Dry matter and ash content in the whole body and filet increased quadratically with increasing salt levels, whereas gill moisture and protein content decreased linearly. Furthermore, the nitrogen, lipid, and energy utilization efficiencies decreased with their respective intake and gain levels. Dietary salt significantly influenced the fatty acid profiles of gill, liver, and filet tissues. In the gill, monounsaturated fatty acids (16:1n-7, ΣMUFA) and n-6 PUFA (20:2n-6) increased, whereas EPA and DHA decreased. Liver ΣSFA (16:0, 18:0) increased, and n-3 PUFA (18:3n-3, 20:5n-3) decreased with increasing dietary salt. Filet saturated fatty acids (14:0, 15:0, 17:0) and n-6 PUFA (20:2n-6, 20:4n-6) increased, while n-3 PUFA (18:3n-3, EPA) decreased with dietary salt. DHA levels in filets showed a quadratic increase. Overall, this study shows that increasing dietary salt negatively impacts turbot growth, feed utilization, and tissue fatty acid composition in brackish water, highlighting the need for further studies on salinity management strategies for turbot aquaculture.

## Introduction

Turbot is a cold-water flatfish species that is widely farmed in Europe and Asia because of its high market demand and adaptability to a wide range of salinity conditions (Person-Le Ruyet 2010; Wuertz et al. 2023). Its global production reached 77,110 tons in 2019 (FAO 2022). Turbot is an euryhaline or relatively stenohaline species that can tolerate a large range of salinities between 5 and 36‰ (Burel et al. 1996; Dietz et al. 2013; Gaumet et al. 1995). Optimum salinity conditions for turbot have been reported to be between 15 and 30‰ for growth and nutrient utilization performance (Dietz et al. 2013; Imsland et al. 2001, 2003). Fish in aquaculture conditions have little chance of sudden and dramatic changes in their environments in terms of salinity, but sometimes water with a salinity out of the optimal range of species can be used (Cech 2000; Liu et al. 2021). Recently, turbot aquaculture has been using less saline water due to the increasing use of deep-well seawater as well as the expansion of farming activities to inland areas, like in southwest Türkiye (Aksoy et al. 2012), Europe (APROMAR 2023; Person-Le Ruyet 2010), and north China (Liu et al. 2021). Therefore, turbot production in the latter areas is generally dependent on underground cold-brackish water with a salinity of 6–15‰ (Liu et al. 2021; Wang and Xu 2019; Wuertz et al. 2023). Additionally, using low-salinity waters in recirculation aquaculture systems can be advantageous because of less dependence on artificial salts (Alam et al. 2021). However, under such conditions, fish must cope with salinity stress and expend extra energy for osmoregulation, which may result in lower growth rates and feed conversion efficiency (Dietz et al. 2013; Imsland et al. 2003).

Osmoregulation is a vital physiological function for fish to maintain a stable intracellular concentration of ions in environments with variable salinity (Cech 2000). Fish osmoregulate by actively transporting ions across their gills, skin, and intestines, which require energy (Cech 2000; NRC 2011). They minimize the energy requirement of osmoregulation by seeking a medium that is isotonic with their body fluids in nature (Febry and Lutz 1987; Kidder et al. 2006). Under aquaculture conditions, euryhaline fish perform better in waters that fit their specific optimal salinity ranges (Dietz et al. 2013; Lall 2022; NRC 2011). Considering that turbot performs better when reared at intermediate salinity (ca. 20 g/L) (Imsland et al. 2001), underground waters with lower salinity could reduce growth and nutrient utilization performance. Oral administration of salt sources can be an option in hyposaline waters since the addition of salt to the diet of fish may increase the absorption of amino acids and provide ions that fish cannot extract from hypotonic environments for whole-body Na^+^ and Cl^−^ homeostasis, thus saving energy expenditure (Salman 2009).

Dietary salt supplementation has increased the success of seawater adaptation by salmonids (Araujo et al. 2023; Duston 1993; Hanson et al. 2016; Pellertier and Besner 1992; Salman 2009; Salman and Eddy 1990; Staurnes and Finstad 2000; Zaugg et al. 1983). Growth and feed efficiency in several fish species, including Asian sea bass, *Lates calcarifer*, European seabass, *Dicentrachus labrax*, gilthead seabream, *Sparus aurata*, common carp, *Cyprinus carpio*, red drum, *Sciaenops ocellatus*, Nile tilapia, *Oreochromis niloticus*, and black sea bass, *Centropristis striata*, can be improved using moderately salt-enriched diets (%1–5) in freshwater and brackish water, potentially due to osmoregulatory advantages and the sparing of energy used in osmoregulation (Alam et al. 2015; Alam et al. 2021; Appelbaum and Arockiaraj 2009; Eroldoğan et al. 2005; Fontaínhas-Fernandes et al. 2000; Gangadhara et al. 2004; Gatlin et al. 1992; Harpaz et al. 2005). However, studies conducted in full-strength seawater indicate that the dietary inclusion of salt does not improve the growth and nutrient utilization of fish (Eroldoğan et al. 2005; Gatlin et al. 1992; Salman and Eddy 1990). This background suggests that dietary salt addition may enhance growth performance and nutrient retention in turbot maintained in brackish waters by reducing the energy expenditure for osmoregulation.

Water salinity has a significant effect on the metabolism and composition of fatty acids in fish and, in general, aquatic organisms (Gan et al. 2016; Hunt et al. 2011; Marrero et al. 2024; Tocher et al. 1995; Xie et al. 2015). In the case of Nile tilapia, Gan et al. (2016) found that only the whole body lipid content decreased with increasing salinity, indicating the potentially important role of lipids and fatty acids in the osmoregulation of fish. The decrease in salinity caused an increase in the percentages of 18:1n-9, 24:1n-9, 18:3n-3, and 18:2n-6, and a decrease in the percentages of 14:0, as observed in European sea bass (Hunt et al. 2011). Takeuchi et al. (1989) found that the total lipid of gills decreased from 897 mg of the initial value to 727 and 747 mg after adaptation to 1% or 2% salt water for 24 h. Additionally, Tocher et al. (1995) studied the effects of salinity on the fatty acid compositions of total lipid and individual glycerophospholipid classes of Atlantic salmon, *Salmo salar*, and turbot cells in culture and found that the most dramatic effects of salinity on total lipid fatty acids were observed in polyunsaturated fatty acids (PUFA) in turbot cells. Yet, the influence of oral salt administration on the fatty acid profiles of tissues remains unknown in turbot.

In fact, although a vast amount of information exists on the relationship between water salinity and fatty acid profiles of fish, such information, e.g., the influence of dietary salt levels on fatty acid compositions of fish tissues, is highly scarce. Chinook salmon, *Oncorhynchus tshawytscha*, fed diets with supplemental salt for 6 weeks showed significant changes in their fatty acid compositions, particularly 16:0, 18:2n-6, and n-3 long-chain polyunsaturated fatty acids (n-3 LC-PUFAs), primarily eicosapentaenoic acid (EPA, 20:5n-3) and docosahexaenoic acid (DHA, 22:6n-3), compared with a control diet (Hanson et al. 2016). Exploring the influence of dietary salt on growth and tissue fatty acid profiles in turbot will be significant, considering that the expansion of turbot aquaculture will require more dilute cold waters (Aksoy et al. 2011; APROMAR 2023; Liu et al. 2021; Person-Le Ruyet 2010).

Therefore, the aim of this study was to assess the influence of different levels of dietary salt on the growth dynamics and feed utilization efficacy and the whole-body nutrient profile and tissue fatty acid compositions (gill, liver, and fillet) of turbot in brackish water.

## Materials and methods

### Fish and rearing conditions

The fish used in the study were selected from a large population with an initial average weight of 120.3 ± 0.03 g. Twenty fish were randomly distributed in experimental tanks with a volume of 140 L. The tanks were connected to a flow-through system running with UV-treated brackish water, and the water flow rate to each tank was 2.5 L/min. Over the study period, water temperature, dissolved oxygen, pH, and salinity were monitored daily using a hand DO meter and pH meter (YSI Model 55 and 63, YSI Inc., Yellowsprings, OH, USA). The average water temperature was 19.7 ± 1.4 °C, dissolved oxygen 7.3 ± 0.3 mg/L, pH 7.9 ± 0.1, and salinity: 8.6 ± 0.1‰, respectively. A natural photoperiod changing between 11.5–12 h light:12–12.5 h dark during a 9-week study period was used. During the study, fish in each tank were bulk weighed at 3-week intervals, and at the end of the experiment after about a 1-day starvation period under anesthesia with ethylene glycol monophenyl ether (0.3 mL/L).

At the beginning of the study, 15 fish were sampled for initial body composition, whereas at the end of the experiment, four fish from each tank were sacrificed with an overdose of ethylene glycol monophenyl ether (1.2 mL/L) to determine the final proximate composition, and the remaining four were used for organo-somatic indices. The organs, including the liver, gill, and filet of the later samples, were separated to determine their proximate and fatty acid compositions.

### Experimental diets and feeding

A commercial extruded diet with a diameter of 9 mm (55% protein and 12% lipid) was coated with predetermined salt solutions (with ultra-pure water). Sodium chloride (Merck, Germany) was used as a dietary salt supplement at concentrations of 0%, (control) 1 (S1), 2 (S2), 4 (S4), and 6% (S6). The salt was first dissolved in ultrapure water and then sprayed onto the pellets and allowed to dry at room temperature for 48 h with occasional mixing. Only water was applied to the control diet. The analyzed dietary salt levels were 1.8, 3.0, 3.6, 5.3, and 6.4%, respectively (Table 1). Fish were fed their respective diets until apparent satiation by hand at 09:00 and 15:30 h.

**Table 1:** Crude nutrient (dry matter basis), salt, and fatty acid profiles of experimental diets top-coated with salt (%)

	Control	S1	S2	S4	S6
Dry matter	93.47	93.09	92.85	92.06	94.05
Protein	56.07	55.53	54.50	51.97	51.58
Lipid	13.88	12.83	13.61	13.24	13.27
Ash	9.45	10.52	11.12	12.74	14.08
Gross energy (MJ/kg)	22.32	21.86	21.86	21.34	21.09
Analyzed salt	1.83	2.96	3.57	5.29	6.35
Fatty acids					
14:0	5.03	5.07	5.17	5.20	4.90
15:0	0.34	0.35	0.35	0.35	0.34
16:0	13.66	13.78	13.94	13.87	13.59
17:0	0.36	0.36	0.36	0.35	0.36
18:0	2.86	2.90	2.95	2.97	2.91
20:0	0.31	0.30	0.30	0.30	0.31
21:0	0.19	0.19	0.18	0.18	0.18
∑SFA	22.98	23.18	23.44	23.39	22.82
16:1n-7	5.25	5.20	5.29	5.30	5.16
18:1n(9 + 7)	23.60	23.77	23.63	23.62	23.44
20:1	3.56	3.53	3.50	3.44	3.54
22:1n-9	0.45	0.45	0.44	0.43	0.46
∑MUFA	32.96	33.05	32.91	32.81	32.66
18:2n-6	8.71	8.85	8.61	8.68	8.57
18:3n-3	2.36	2.39	2.37	2.32	2.33
20:2n-6	0.45	0.45	0.44	0.43	0.45
20:4n-6	0.52	0.51	0.51	0.51	0.53
20:5n-3	8.75	8.57	8.75	8.80	8.89
22:6n-3	8.22	8.21	8.45	8.32	8.64
∑PUFA	29.20	29.16	29.27	29.18	29.58
∑n6	9.86	9.98	9.71	9.74	9.72
∑n3	19.33	19.18	19.56	19.44	19.86
n6/n3	0.51	0.52	0.50	0.50	0.49
DHA/EPA	0.94	0.96	0.97	0.95	0.97
n3/n6	1.96	1.92	2.01	2.00	2.04
n-3 Lc PUFA	16.98	16.78	17.20	17.12	17.53
Lc PUFA	18.13	17.92	18.30	18.18	18.68
TOTAL	85.14	85.39	85.62	85.37	85.06
PUFA/SFA	1.27	1.26	1.25	1.25	1.30
Others	14.86	14.61	14.38	14.63	14.94

∑SFA = 12:0 + 14:0 + 15:0 + 16:0 + 17:0 + 18:0 + 20:0 + 21:0 + 22:0; ∑MUFA = 14:1 + 15:1 + 6:1n-7 + 17:1 + 18:1n(9 + 7) + 20:1 + 22:1n-9; ∑PUFA = 18:2n-6 + 18:3n-3 + 20:2n-6 + 20:3n-6 + 20:4n-6 + 20:5n-3 + 22:6n-3 + 22:2n-6; n-3 LC PUFA = 20:5n-3 + 22:6n-3; Lc PUFA = 20:5n-3 + 22:6n-3 + 20:2n-6 + 20:3n-6 + 20:4n-6 + 22:2n-6

### Chemical analysis

Fish samples were stored at − 20 °C until analysis. Prior to analysis, the samples were thawed in a refrigerator overnight and then homogenized using a kitchen meat chopper (Tefal Le Hachoir 1500, France), except for liver and gill tissue, which were finely chopped using a stainless steel knife. Proximate analysis, except for crude lipid, of experimental diets and fish was performed according to the methods of AOAC (1990): dry matter at 104 °C until constant weight, ash content by incineration in a muffle furnace at 600 °C for 2 h; crude protein (N × 6.25) by the Kjeldhal method after acid digestion. Crude lipid was determined with ether extraction using an automatic extraction system (ANKOMXT15 Extractor, ANKOM Technology, Macedon, USA). For fatty acid profile determination, the lipids of all samples were extracted according to Bligh and Dyer (1959). Fatty acid methyl esters (FAME) were prepared following Ichihara et al. (1996). The fatty acids were analyzed by a gas chromatography (GC) (Focus GC, Thermo Electron, Waltham, MA) equipped with an autosampler, a flame ionization detector, and a fused silica capillary column (30 m × 0.32 mm, ID × 0.25 μm film). The oven temperature was set at 140 °C for 5 min, raised to 200 °C at a rate of 4 °C/min, and then 220 °C at a rate of 1 °C/min, while the injector and detector temperatures were set at 220 °C and 280 °C, respectively. FAMEs were identified by comparing the retention times with those of the SUPELCO standard (Sigma-Aldrich). The salt concentrations of the experimental diets were determined by the titrimetric method (James 2013).

### Statistical analysis

Linear and quadratic polynomial contrasts were used to detect the linear and quadratic effects of various dietary salt levels on the variables at a level of *P* < 0.10 using the GLM procedure in JMP software (version 8, SAS Institute Inc., Cary, NC). A linear-quadratic nonlinear model was developed to estimate the maximum tolerable dietary salt level based on specific growth rate (SGR) values. In the model, the slope of the linear part was set to 0, while the slopes of the quadratic part were forced to get values below 0. The model was run on GraphPad Prism 7.

## Results

**Fig. 1:**
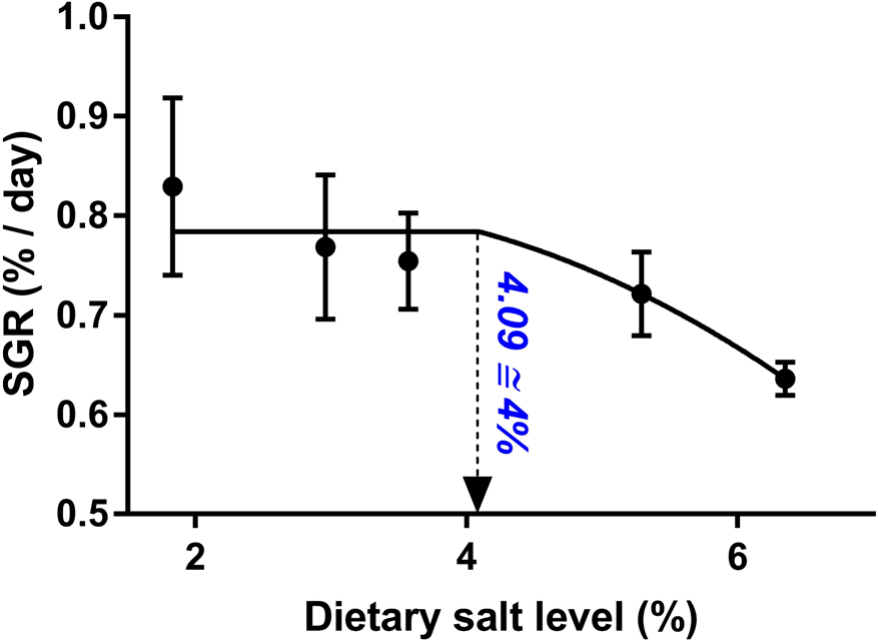
Relationship between dietary salt levels and SGR of turbot fed experimental diets for 9 weeks. Error bars represent standard error of means of three replications

Juvenile turbot responded to increased dietary salt levels with significant deteriorations in FW, SGR, and FCR (Table 2). The nonlinear model showed that turbot can tolerate dietary levels up to a maximum of approximately 4% (Fig. 1). However, there was no significant change in dietary feed intake (DFI) and protein efficiency ratio (PER) in response to the treatments. Body indices, including condition factor (CF) and hepato somatic index (HSI), linearly decreased with the increase of dietary salt concentrations, whereas no alteration in VSI values occurred. The nutrient utilization variables are given in Table 3. Changes in nitrogen, lipid, and energy utilization variables were mostly related to their intake and gain levels, with a significant linear decrease (*P* < 0.10).

**Table 2:** Effects of dietary salt concentrations on growth performance, nutrient utilization, and body indices in turbot

Fatty acids	Control	S1	S2	S4	S6*	*P* values	
						Linear	Quadratic
IW^1^	120.3 ± 0.1	120.3 ± 0.1	120.4 ± 0.1	120.3 ± 0.0	120. 4 ± 0.1	-	-
FW^2^	188.6 ± 78.79	182.47 ± 7.24	181.10 ± 4.84	177.73 ± 4.06	169.7 ± 1.4	**0.015**	0.917
SGR^3^	0.83 ± 0.09	0.77 ± 0.07	0.75 ± 0.05	0.72 ± 0.04	0.64 ± 0.02	**0.016**	0.873
DFI (g/kg MBW^0.8^)^4^	4.32 ± 0.35	4.41 ± 0.14	4.06 ± 0.19	3.91 ± 0.16	4.13 ± 0.04	0.125	0.464
FCR^5^	0.76 ± 0.03	0.85 ± 0.06	0.79 ± 0.02	0.79 ± 0.02	0.95 ± 0.02	**0.018**	0.209
PER^6^	2.36 ± 0.09	2.15 ± 0.17	2.34 ± 0.06	2.43 ± 0.07	2.03 ± 0.04	0.382	0.285
CF^7^	1.48 ± 0.02	1.47 ± 0.04	1.44 ± 0.02	1.43 ± 0.01	1.41 ± 0.01	**0.012**	0.818
VSI^8^	4.79 ± 0.13	4.92 ± 0.04	4.67 ± 0.09	4.64 ± 0.21	4.62 ± 0.14	0.135	0.929
HSI^9^	1.22 ± 0.13	1.37 ± 0.04	1.17 ± 0.02	1.18 ± 0.11	1.02 ± 0.03	**0.046**	0.172

^1^Initial weight (g)

^2^Final weight (g)

^3^Specific growth rate = 100 × [(ln FW − ln IW)/days]

^4^Daily feed intake (DFI g/kg MBW/day) = (dry matter intake/MBW^0.8^)/day, where metabolic body weight (MBW) = (geometric mean of IW and FW)^0.8^

^5^Feed conversion ratio = (dry feed intake (g) − filler intake (g))/wet weight gain (g)

^6^Protein efficiency ratio = weight gain (g)/protein fed (g)

^7^Condition factor = 100 × (body weight (g)/total length^3^ (cm)

^8^Viscero somatic index = 100 × (visceral weight (g)/body weight (g))

^9^Hepato somatic index = 100 × (liver weight (g)/body weight (g))

**Table 3:** Effects of dietary salt concentrations on nutrient balances of turbot

	Control	S1	S2	S4	S6	*P* values	
						Linear	Quadratic
*Nitrogen*							
Intake (g/kg/MBW^0.8^/day)	0.39 ± 0.03	0.39 ± 0.01	0.35 ± 0.02	0.32 ± 0.01	0.34 ± 0.00	**0.016**	0.551
Gain (g/kg/MBW^08^/day)	0.18 ± 0.03	0.16 ± 0.01	0.16 ± 0.01	0.15 ± 0.02	0.13 ± 0.01	**0.038**	0.936
Retention (%)	45.49 ± 3.33	38.11 ± 2.93	45.49 ± 3.33	38.11 ± 2.93	45.49 ± 3.33	0.400	0.584
*Lipid*							
Intake (g/kg/MBW^0.8^/day)	0.60 ± 0.05	0.57 ± 0.02	0.55 ± 0.03	0.52 ± 0.02	0.55 ± 0.01	**0.033**	0.178
Gain (g/kg/MBW^0.8^/day)	0.30 ± 0.04	0.22 ± 0.06	0.25 ± 0.04	0.22 ± 0.02	0.22 ± 0.02	0.121	0.425
Retention (%)	48.85 ± 2.77	39.24 ± 8.69	45.66 ± 5.68	43.05 ± 2.74	40.58 ± 4.05	0.325	0.677
*Energy*							
Intake (kJ/kg/MBW^0.8^/day)	96.37 ± 7.81	96.36 ± 3.14	88.78 ± 4.09	83.37 ± 3.47	87.06 ± 0.95	**0.018**	0.434
Gain (kJ/kg/MBW^0.8^/day)	33.69 ± 4.69	28.36 ± 3.94	29.56 ± 3.07	27.32 ± 2.84	24.97 ± 1.89	**0.049**	0.683
Retention (%)	34.61 ± 2.38	29.26 ± 3.30	33.13 ± 1.93	32.60 ± 1.97	28.65 ± 1.88	0.222	0.963

The whole body, filet, viscera, liver, and gill proximate compositions are displayed in Table 4. Dietary increasing salt concentrations quadratically affected the whole body dry matter and ash and filet ash levels of turbot. There were no significant changes in the visceral and liver nutrient compositions, whereas gill dry matter and protein levels linearly reduced with the increasing salt levels.

**Table 4:** Effects of dietary salt concentrations on the proximate compositions (%) of whole body and selected organs

Fatty acids	Control	S1	S2	S4	S6*	*P* values	
						Linear	Quadratic
Whole body							
Dry matter	23.30 ± 0.50	22.31 ± 0.12	22.63 ± 0.44	22.43 ± 0.34	22.61 ± 0.22	0.117	**0.099**
Protein	15.39 ± 0.29	15.16 ± 0.08	15.26 ± 0.12	15.21 ± 0.29	15.19 ± 0.35	0.537	0.694
Lipid	2.51 ± 0.17	2.11 ± 0.29	2.33 ± 0.23	2.16 ± 0.12	2.23 ± 0.14	0.239	0.332
Ash	4.29 ± 0.06	4.20 ± 0.12	4.13 ± 0.04	4.19 ± 0.11	4.28 ± 0.05	0.740	**0.076**
Filet							
Dry matter	23.66 ± 0.22	23.29 ± 0.22	23.04 ± 0.17	23.29 ± 0.35	23.29 ± 0.27	0.227	0.122
Protein	19.15 ± 0.26	19.00 ± 0.17	19.33 ± 0.31	19.27 ± 0.13	19.25 ± 0.28	0.366	0.232
Lipid	2.50 ± 0.21	2.24 ± 0.10	2.09 ± 0.21	2.07 ± 0.28	2.14 ± 0.37	0.141	0.266
Ash	1.67 ± 0.04	1.58 ± 0.04	1.50 ± 0.06	1.63 ± 0.06	1.65 ± 0.10	0.947	**0.064**
Viscera							
Dry matter	16.23 ± 0.64	16.44 ± 0.12	16.47 ± 0.71	16.44 ± 0.12	16.77 ± 0.12	0.373	0.948
Protein	12.57 ± 0.64	12.73 ± 0.25	13.21 ± 0.93	12.98 ± 0.34	13.44 ± 0.31	0.218	0.889
Lipid	0.99 ± 0.10	1.08 ± 0.14	0.87 ± 0.07	0.90 ± 0.05	0.96 ± 0.06	0.370	0.548
Ash	1.36 ± 0.23	1.22 ± 0.05	1.14 ± 0.05	1.18 ± 0.01	1.37 ± 0.21	0.622	0.113
Liver							
Dry matter	23.24 ± 0.66	25.04 ± 1.14	22.98 ± 0.46	24.63 ± 1.40	23.85 ± 0.17	0.558	0.578
Protein	11.43 ± 1.20	11.11 ± 0.49	11.46 ± 0.49	12.14 ± 1.24	11.28 ± 0.73	0.709	0.782
Lipid	4.22 ± 0.89	4.46 ± 1.21	4.01 ± 0.72	4.48 ± 1.50	4.90 ± 0.67	0.633	0.726
Gill							
Dry matter	23.50 ± 0.30	22.56 ± 0.09	23.28 ± 0.20	22.25 ± 0.44	22.50 ± 0.53	**0.023**	0.467
Protein	15.75 ± 0.10	14.91 ± 0.37	14.74 ± 0.84	14.71 ± 0.21	14.80 ± 0.38	**0.065**	0.136
Lipid	0.43 ± 0.01	0.48 ± 0.03	0.44 ± 0.06	0.42 ± 0.01	0.47 ± 0.05	0.891	0.967
Ash	6.38 ± 0.24	6.02 ± 0.79	6.31 ± 0.16	6.09 ± 0.24	6.30 ± 0.29	0.813	0.609

The effects of dietary salt concentrations on gill fatty acid profiles are shown in Table 5. Although there was no significant change in saturated fatty acids, the levels of 16:1n-7 and total monounsaturated fatty acids (∑MUFA) linearly increased with dietary salt levels (*P* < 0.10). There was a quadratic linear increase in 20:2n-6 but a linear decrease in 20:4n-6 in response to dietary salt concentrations. The gill EPA and DHA levels linearly decreased with increasing dietary salt levels. The liver total saturated fatty acids (∑SFA) linearly increased with salt levels, mainly due to increases in 16:0 and 18:0 (Table 6). There was no significant change in monounsaturated and n-6 fatty acid groups, whereas 18:3n-3 and 20:5n-3 levels linearly decreased with increasing salt intakes (*P* < 0.10). The filet fatty acid compositions were significantly affected by dietary treatments (*P* < 0.10). (Table 7). While 14:0, 15:0, and 17:0 levels linearly increased with dietary salt levels, there was no significant change in monounsaturated fatty acids. The effect of dietary salt levels on 20:2n-6 and 20:4n-6 compositions was a significant increase. The filet 18:3n-3 and EPA levels linearly reduced with salt levels, but a quadratic linear increase occurred in the DHA levels. However, the whole body fatty acid profiles of fish did not significantly change with increasing dietary salt concentrations (Table 8).

**Table 5:** Effects of dietary salt concentrations on the gill fatty acid compositions (%) of turbot

Fatty acids	Control	S1	S2	S4	S6*	*P* values	
						Linear	Quadratic
14:0	4.07 ± 0.17	4.38 ± 0.26	4.11 ± 0.26	4.13 ± 0.01	4.42 ± 0.20	0.387	0.950
15:0	0.43 ± 0.02	0.44 ± 0.01	0.44 ± 0.02	0.44 ± 0.02	0.46 ± 0.02	0.192	0.709
16:0	18.68 ± 0.83	18.36 ± 0.30	18.85 ± 0.78	18.38 ± 0.67	19.01 ± 0.40	0.790	0.561
17:0	0.53 ± 0.01	0.51 ± 0.01	0.54 ± 0.02	0.50 ± 0.01	0.54 ± 0.01	0.750	0.184
18:0	4.78 ± 0.31	4.65 ± 0.06	4.61 ± 0.00	4.53 ± 0.11	4.80 ± 0.26	0.694	0.265
20:0	0.33 ± 0.01	0.31 ± 0.01	0.34 ± 0.03	0.33 ± 0.01	0.36 ± 0.02	0.274	0.196
21:0	0.26 ± 0.01	0.28 ± 0.01	0.29 ± 0.03	0.29 ± 0.01	0.28 ± 0.03	0.156	0.265
∑SFA	29.13 ± 1.19	29.03 ± 0.58	29.25 ± 0.95	28.68 ± 0.74	29.95 ± 0.65	0.677	0.481
16:1n-7	4.49 ± 0.10	4.65 ± 0.10	4.64 ± 0.21	4.72 ± 0.10	4.78 ± 0.17	**0.064**	0.634
18:1n(9 + 7)	25.65 ± 0.39	25.82 ± 0.14	26.31 ± 0.28	26.16 ± 0.72	25.78 ± 0.05	0.311	0.125
20:1	3.04 ± 0.05	3.04 ± 0.08	3.15 ± 0.17	3.14 ± 0.03	3.12 ± 0.08	0.217	0.575
22:1n-9	0.38 ± 0.02	0.39 ± 0.03	0.42 ± 0.05	0.41 ± 0.01	0.41 ± 0.02	0.309	0.563
∑MUFA	33.58 ± 0.43	33.93 ± 0.29	34.55 ± 0.25	34.47 ± 0.81	34.13 ± 0.20	**0.067**	0.111
18:2n-6	9.46 ± 0.24	9.57 ± 0.12	9.42 ± 0.02	9.72 ± 0.20	9.35 ± 0.22	0.952	0.392
18:3n-3	1.35 ± 0.10	1.40 ± 0.03	1.36 ± 0.01	1.44 ± 0.03	1.33 ± 0.05	0.774	0.299
20:2n-6	0.88 ± 0.01	0.95 ± 0.01	0.97 ± 0.05	0.94 ± 0.03	0.93 ± 0.01	**0.026**	**0.003**
20:4n-6	0.76 ± 0.04	0.73 ± 0.04	0.70 ± 0.01	0.66 ± 0.04	0.64 ± 0.05	**0.008**	0.473
20:5n-3	4.43 ± 0.17	4.44 ± 0.16	4.11 ± 0.07	4.20 ± 0.49	4.04 ± 0.06	**0.077**	0.946
22:6n-3	8.75 ± 0.53	8.30 ± 0.34	8.28 ± 0.21	8.07 ± 0.23	7.68 ± 0.40	**0.031**	0.852
∑PUFA	25.79 ± 0.57	25.53 ± 0.61	24.95 ± 0.10	25.21 ± 0.65	24.13 ± 0.24	**0.020**	0.707
∑n6	11.25 ± 0.17	11.39 ± 0.16	11.20 ± 0.19	11.50 ± 0.11	11.08 ± 0.15	0.823	0.226
∑n3	14.53 ± 0.59	14.14 ± 0.47	13.75 ± 0.29	13.71 ± 0.75	13.05 ± 0.36	**0.019**	0.965
n6/n3	0.78 ± 0.04	0.81 ± 0.02	0.82 ± 0.03	0.84 ± 0.05	0.85 ± 0.03	**0.039**	0.682
DHA/EPA	1.98 ± 0.13	1.87 ± 0.06	2.01 ± 0.02	1.94 ± 0.17	1.90 ± 0.10	0.695	0.980
n-3 LcPUFA	13.18 ± 0.59	12.74 ± 0.46	12.39 ± 0.28	12.27 ± 0.72	11.72 ± 0.41	**0.018**	0.860
∑LcPUFA	14.97 ± 0.66	14.56 ± 0.53	14.18 ± 0.11	14.05 ± 0.82	13.45 ± 0.49	**0.027**	0.935
n3/n6	1.29 ± 0.06	1.24 ± 0.03	1.23 ± 0.05	1.19 ± 0.08	1.18 ± 0.05	**0.039**	0.632

∑SFA = 12:0 + 14:0 + 15:0 + 16:0 + 17:0 + 18:0 + 20:0 + 21:0 + 22:0; ∑MUFA = 14:1 + 15:1 + 6:1n-7 + 17:1 + 18:1n(9 + 7) + 20:1 + 22:1n-9; ∑PUFA = 18:2n-6 + 18:3n-3 + 20:2n-6 + 20:3n-6 + 20:4n-6 + 20:5n-3 + 22:6n-3 + 22:2n-6; n-3 LcPUFA = 20:5n-3 + 22:6n-3; LcPUFA = 20:5n-3 + 22:6n-3 + 20:2n-6 + 20:3n-6 + 20:4n-6 + 22:2n-6

**Table 6:** Effects of dietary salt concentrations on the liver fatty acid compositions (%) of turbot

Fatty acids	Control	S1	S2	S4	S6*	*P* values	
						Linear	Quadratic
14:0	4.67 ± 1.05	5.60 ± 0.09	5.05 ± 0.13	5.04 ± 0.76		0.582	0.399
15:0	0.45 ± 0.03	0.48 ± 0.05	0.45 ± 0.03	0.44 ± 0.03		0.997	0.496
16:0	15.22 ± 1.28	18.70 ± 2.50	18.23 ± 0.65	19.05 ± 0.44		**0.039**	0.220
17:0	0.62 ± 0.06	0.58 ± 0.09	0.76 ± 0.06	0.61 ± 0.10		0.724	0.524
18:0	4.56 ± 0.55	4.35 ± 0.79	5.26 ± 0.54	6.62 ± 0.71		**0.035**	0.289
20:0	0.22 ± 0.02	0.24 ± 0.03	0.22 ± 0.02	0.27 ± 0.01		0.127	0.593
21:0	0.29 ± 0.04	0.31 ± 0.07	0.20 ± 0.02	0.27 ± 0.03		0.420	0.589
∑SFA	26.02 ± 1.87	30.26 ± 3.47	30.16 ± 0.26	32.30 ± 0.40		**0.022**	0.382
16:1n-7	4.55 ± 0.74	4.76 ± 0.21	4.52 ± 0.09	4.34 ± 0.76		0.814	0.715
18:1n(9 + 7)	23.83 ± 0.43	24.21 ± 1.10	24.23 ± 1.86	22.83 ± 1.54		0.641	0.434
20:1	3.00 ± 0.39	3.89 ± 0.66	2.83 ± 0.53	3.29 ± 0.33		0.127	0.593
22:1n-9	0.42 ± 0.04	0.53 ± 0.11	0.40 ± 0.05	0.51 ± 0.07		0.477	0.912
∑MUFA	31.80 ± 0.70	33.39 ± 1.91	31.97 ± 1.20	30.97 ± 2.47		0.804	0.412
18:2n-6	9.83 ± 0.90	9.89 ± 0.70	10.15 ± 0.25	9.30 ± 0.40		0.687	0.474
18:3n-3	1.25 ± 0.11	1.07 ± 0.09	1.03 ± 0.04	0.91 ± 0.15		**0.028**	0.541
20:2n-6	1.92 ± 0.29	1.76 ± 0.07	1.78 ± 0.04	1.68 ± 0.24		0.312	0.760
20:4n-6	1.01 ± 0.07	0.75 ± 0.11	1.09 ± 0.09	1.11 ± 0.22		0.423	0.383
20:5n-3	4.61 ± 0.30	3.10 ± 0.38	3.87 ± 0.43	3.86 ± 0.23		**0.067**	**0.026**
22:6n-3	10.80 ± 2.05	7.60 ± 0.58	9.81 ± 0.87	11.58 ± 2.99		0.954	0.173
∑PUFA	28.66 ± 1.19	24.18 ± 1.17	27.72 ± 1.63	28.45 ± 3.17		0.784	0.159
∑n6	12.42 ± 0.94	12.41 ± 0.80	13.02 ± 0.30	12.10 ± 0.29		0.933	0.500
∑n3	16.24 ± 1.97	11.77 ± 0.63	14.70 ± 1.33	16.36 ± 3.00		0.800	0.104
n6/n3	0.80 ± 0.15	1.06 ± 0.07	0.89 ± 0.06	0.79 ± 0.14		0.225	0.185
DHA/EPA	2.30 ± 0.30	2.54 ± 0.40	2.54 ± 0.06	2.96 ± 0.69		0.267	0.933
n-3 LC pufa	15.41 ± 2.35	10.70 ± 0.58	13.68 ± 1.30	15.44 ± 3.15		0.774	0.109
LcPUFA	18.01 ± 2.33	13.22 ± 0.60	16.54 ± 1.34	18.24 ± 3.57		0.855	0.137
n3/n6	1.35 ± 0.27	0.96 ± 0.07	1.13 ± 0.08	1.35 ± 0.23		0.700	**0.089**

∑SFA = 12:0 + 14:0 + 15:0 + 16:0 + 17:0 + 18:0 + 20:0 + 21:0 + 22:0; ∑MUFA = 14:1 + 15:1 + 6:1n7 + 17:1 + 18:1n(9 + 7) + 20:1 + 22:1n-9;

∑PUFA = 18:2n-6 + 18:3n-3 + 20:2n-6 + 20:3n-6 + 20:4n- + 20:5n-3 + 22:6n-3 + 22:2n-6;

n-3 LcPUFA = 20:5n-3 + 22:6n-3;

LcPUFA = 20:5n-3 + 22:6n-3 + 20:2n-6 + 20:3n-6 + 20:4n-6 + 22:2n-6

^*^Could not be analyzed due to an accidental drop of sample tubes

**Table 7:** Effects of dietary salt concentrations on filet fatty acid compositions (%) of turbot

Fatty acids	Control	S1	S2	S4	S6*	*P* values	
						Linear	Quadratic
14:0	5.10 ± 0.12	4.99 ± 0.11	5.05 ± 0.05	5.23 ± 0.04	5.26 ± 0.01	**0.032**	0.197
15:0	0.42 ± 0.01	0.41 ± 0.01	0.43 ± 0.00	0.44 ± 0.00	0.44 ± 0.00	**0.017**	0.934
16:0	14.18 ± 0.25	13.97 ± 0.30	14.10 ± 0.16	14.18 ± 0.17	14.53 ± 0.22	0.227	0.162
17:0	0.33 ± 0.01	0.33 ± 0.00	0.34 ± 0.01	0.35 ± 0.01	0.36 ± 0.01	**0.006**	0.394
18:0	2.69 ± 0.08	2.67 ± 0.03	2.77 ± 0.10	2.70 ± 0.04	2.78 ± 0.07	0.407	0.916
20:0	0.23 ± 0.01	0.23 ± 0.00	1.43 ± 1.21	0.22 ± 0.01	0.24 ± 0.01	0.994	0.235
21:0	0.32 ± 0.01	0.35 ± 0.01	0.40 ± 0.05	0.34 ± 0.01	0.34 ± 0.01	0.617	**0.064**
∑SFA	23.36 ± 0.43	23.05 ± 0.41	24.61 ± 1.38	23.51 ± 0.21	24.02 ± 0.28	0.462	0.675
16:1n-7	5.29 ± 0.04	5.26 ± 0.07	5.16 ± 0.08	5.29 ± 0.02	5.32 ± 0.02	0.659	**0.072**
18:1n(9 + 7)	24.28 ± 0.21	24.36 ± 0.05	23.80 ± 0.20	24.00 ± 0.09	24.05 ± 0.21	0.142	0.251
20:1	3.56 ± 0.08	3.55 ± 0.06	3.60 ± 0.02	3.63 ± 0.03	3.65 ± 0.11	0.173	0.962
22:1n-9	0.47 ± 0.01	0.48 ± 0.01	0.46 ± 0.01	0.45 ± 0.01	0.48 ± 0.01	0.706	0.284
∑MUFA	33.82 ± 0.16	33.90 ± 0.03	33.30 ± 0.15	33.53 ± 0.06	33.67 ± 0.23	0.174	0.109
18:2n-6	11.23 ± 0.05	11.24 ± 0.07	11.18 ± 0.06	11.25 ± 0.04	11.15 ± 0.12	0.552	0.798
18:3n-3	1.94 ± 0.06	1.94 ± 0.03	1.82 ± 0.06	1.83 ± 0.02	1.80 ± 0.01	**0.006**	0.553
20:2n-6	0.89 ± 0.02	0.94 ± 0.04	0.97 ± 0.01	0.97 ± 0.03	0.95 ± 0.03	**0.024**	**0.036**
20:4n-6	0.57 ± 0.02	0.57 ± 0.02	0.63 ± 0.03	0.62 ± 0.01	0.60 ± 0.01	**0.055**	0.146
20:5n-3	5.54 ± 0.27	5.53 ± 0.11	5.19 ± 0.10	5.30 ± 0.13	5.25 ± 0.06	**0.087**	0.417
22:6n-3	9.01 ± 0.05	8.96 ± 0.32	10.14 ± 0.37	9.88 ± 0.06	9.50 ± 0.40	**0.049**	**0.067**
∑PUFA	29.34 ± 0.32	29.36 ± 0.35	30.08 ± 0.24	29.98 ± 0.14	29.41 ± 0.50	0.336	**0.086**
∑n6	12.86 ± 0.04	12.93 ± 0.11	12.94 ± 0.06	12.98 ± 0.05	12.85 ± 0.16	0.682	0.209
∑n3	16.48 ± 0.29	16.43 ± 0.24	17.14 ± 0.29	17.00 ± 0.19	16.55 ± 0.35	0.317	**0.100**
n6/n3	0.78 ± 0.01	0.79 ± 0.01	0.76 ± 0.02	0.76 ± 0.01	0.78 ± 0.01	0.330	0.190
DHA/EPA	1.64 ± 0.09	1.62 ± 0.08	1.96 ± 0.09	1.87 ± 0.04	1.81 ± 0.10	**0.041**	0.114
n-3 LC PUFA	14.55 ± 0.22	14.49 ± 0.25	15.32 ± 0.34	15.18 ± 0.18	14.76 ± 0.34	0.162	**0.093**
LCPUFA	16.18 ± 0.24	16.20 ± 0.29	17.07 ± 0.35	16.90 ± 0.17	16.46 ± 0.36	0.125	**0.066**

∑SFA = 12:0 + 14:0 + 15:0 + 16:0 + 17:0 + 18:0 + 20:0 + 21:0 + 22:0; ∑MUFA = 14:1 + 15:1 + 6:1n-7 + 17:1 + 18:1n(9 + 7) + 20:1 + 22:1n-9; ∑PUFA = 18:2n-6 + 18:3n-3 + 20:2n-6 + 20:3n-6 + 20:4n-6 + 20:5n-3 + 22:6n-3 + 22:2n-6; n-3 LcPUFA = 20:5n-3 + 22:6n-3; LcPUFA = 20:5n-3 + 22:6n-3 + 20:2n-6 + 20:3n-6 + 20:4n-6 + 22:2n-6

**Table 8:** Effects of dietary salt concentrations on whole body fatty acid compositions (%) of turbot

Fatty acids	Control	S1	S2	S4	S6	*P* values	
						Linear	Quadratic
14:0	5.61 ± 0.27	5.69 ± 0.42	5.46 ± 0.16	5.64 ± 0.18	5.79 ± 0.20	0.686	0.547
15:0	0.49 ± 0.02	0.53 ± 0.07	0.48 ± 0.01	0.50 ± 0.02	0.51 ± 0.02	0.936	0.838
16:0	17.21 ± 0.71	17.98 ± 1.58	16.98 ± 0.31	17.12 ± 0.15	17.72 ± 0.76	0.931	0.835
17:0	0.46 ± 0.02	0.50 ± 0.06	0.44 ± 0.01	0.46 ± 0.01	0.46 ± 0.03	0.869	0.923
18:0	3.57 ± 0.19	3.73 ± 0.38	3.48 ± 0.20	3.58 ± 0.08	3.55 ± 0.07	0.812	0.960
20:0	0.32 ± 0.02	0.32 ± 0.03	0.30 ± 0.01	0.31 ± 0.00	0.31 ± 0.01	0.701	0.715
21:0	0.37 ± 0.01	0.35 ± 0.03	0.35 ± 0.02	0.37 ± 0.01	0.34 ± 0.01	0.408	0.901
∑SFA	26.47 ± 2.63	25.80 ± 4.26	24.18 ± 2.16	28.28 ± 0.36	27.05 ± 2.43	0.597	0.628
16:1n-7	4.48 ± 0.12	4.31 ± 0.32	4.56 ± 0.09	4.56 ± 0.04	4.51 ± 0.18	0.578	0.981
18:1n(9 + 7)	24.50 ± 0.39	23.73 ± 0.90	24.52 ± 0.09	24.46 ± 0.54	23.86 ± 0.89	0.743	0.862
20:1	4.42 ± 0.09	4.44 ± 0.16	4.22 ± 0.02	4.42 ± 0.07	4.26 ± 0.05	0.320	0.824
22:1n-9	0.66 ± 0.03	0.64 ± 0.02	0.62 ± 0.01	0.64 ± 0.02	0.62 ± 0.01	0.175	0.629
∑MUFA	34.13 ± 0.42	33.27 ± 0.95	33.99 ± 0.01	34.14 ± 0.51	33.34 ± 0.99	0.712	0.893
18:2n-6	9.95 ± 0.33	9.86 ± 0.30	10.04 ± 0.07	9.91 ± 0.04	10.02 ± 0.37	0.851	0.922
18:3n-3	1.46 ± 0.08	1.40 ± 0.16	1.50 ± 0.06	1.43 ± 0.03	1.42 ± 0.08	0.803	0.807
20:2n-6	1.03 ± 0.02	1.00 ± 0.02	0.99 ± 0.02	1.03 ± 0.01	0.99 ± 0.01	0.350	0.364
20:4n-6	0.51 ± 0.04	0.56 ± 0.04	0.52 ± 0.02	0.51 ± 0.02	0.51 ± 0.01	0.584	0.429
20:5n-3	3.36 ± 0.15	3.32 ± 0.38	3.61 ± 0.20	3.36 ± 0.04	3.34 ± 0.06	0.976	0.478
22:6n-3	8.12 ± 0.27	8.70 ± 0.28	8.48 ± 0.16	8.21 ± 0.16	8.44 ± 0.18	0.728	0.307
∑PUFA	24.69 ± 0.83	25.05 ± 0.70	25.38 ± 0.48	24.72 ± 0.08	24.95 ± 0.29	0.877	0.473
PUFA/SFA	0.96 ± 0.12	1.03 ± 0.17	1.07 ± 0.11	1.02 ± 0.08	0.94 ± 0.09	0.985	0.289
∑n6	11,71 ± 0,39	11,62 ± 0,30	11,79 ± 0,07	11,72 ± 0,02	11,76 ± 0,36	0.811	0.978
∑n3	12,94 ± 0,49	13,42 ± 0,45	13,60 ± 0,42	13,00 ± 0,10	13,19 ± 0,07	0.880	0.254
n6/n3	0,91 ± 0,01	0,87 ± 0,02	0,87 ± 0,02	0,90 ± 0,01	0,89 ± 0,03	0.971	0.192
DHA/EPA	2.42 ± 0.03	2.72 ± 0.41	2.36 ± 0.09	2.45 ± 0.08	2.53 ± 0.09	0.978	0.983
n-3LCPUFA	11.48 ± 0.42	12.02 ± 0.34	12.10 ± 0.36	11.57 ± 0.12	11.77 ± 0.15	0.806	0.216
LCPUFA	13.24 ± 0.49	13.78 ± 0.33	13.84 ± 0.36	13.38 ± 0.15	13.52 ± 0.16	0.775	0.215

∑SFA = 12:0 + 14:0 + 15:0 + 16:0 + 17:0 + 18:0 + 20:0 + 21:0 + 22:0; ∑MUFA = 14:1 + 15:1 + 6:1n-7 + 17: 221 + 18:1n(9 + 7) + 20:1 + 22:1n-9; ∑PUFA = 18:2n-6 + 18:3n-3 + 20:2n-6 + 20:3n-6 + 20:4n-6 + 20:5n-3 + 22:6n-3 + :2n-6; n-3 LcPUFA = 20:5n-3 + 22:6n-3; LcPUFA = 20:5n-3 + 22:6n-3 + 20:2n-6 + 20:3n-6 + 20:4n-6 + 22:2n-6

## Discussion

In diluted water, fish must meet the requirement for ions like Na^+^, Cl^−^, K^+^, Mg^+^, and Ca^2+^ from their diets because of the passive outward flux of ions (Gatlin et al. 1992; Salman 2009; Shaw et al. 1975; Smith et al. 1995; Tavares-Dias 2022). Therefore, euryhaline saltwater fish should be fed diets supplemented with salt when cultured in suboptimal salinity waters (Salman 2009; Wood and Bucking 2010). The results presented here showed that increasing dietary salt levels led to significant deteriorations in FW, SGR, and FCR, whereas no significant change was observed in DFI and PER, implying that high dietary salt levels may harm the growth and feed efficiency of juvenile turbot. These findings are inconsistent with some studies showing better growth performance in fish when maintained in diluted waters. For instance, red drum fed a 2% NaCI-supplemented diet showed better growth performance and feed efficiency when reared in freshwater and brackish waters but not in full-strength seawater (Gatlin et al. 1992). Dietary salt supplementation of up to 9% significantly increased growth and feed utilization performance in freshwater-adapted European seabass (Eroldoğan et al. 2005). Similarly, Asian seabass displayed an improved FCR response to dietary salt inclusion of up to 4% in freshwater, which was not the case in seawater (Harpaz et al. 2005). Parallel findings were also reported for hybrid tilapia (*Oreochromis niloticus* × *O. aureus*) by Shiau and Lu (2004), who showed that the experimental fish showed a dietary requirement of approximately 1.5 g/kg for sodium in fresh water but not in seawater due to the uptake of sodium from the water. Improvements in FW, feed conversion ratio, and PER in freshwater fish species such as rohu, *Labeo rohita*, mrigal, *Cirrhinus mrigala*, common carp, and hybrid tilapia were also observed when fish were fed diets including salt 1.0–3.0% (Cnaani et al. 2010; Gangadhara et al. 2004; Keshavanath et al. 2003). The inclusion of 5% salt in the diet of Nile tilapia significantly improved the digestibility coefficients for dry matter, protein, lipid, and ash (Hallali et al. 2018). The negative influence of increasing dietary salt content on HSI and CF values could partly explain the cause of poor growth and feed utilization due to several physiological and metabolic disruptions and increased energy demand for osmoregulation.

The influence of dietary salt on growth and feed efficiency was not always positive, as in this study (Table 2 and Fig. 1). For instance, the growth performance and feed efficiency of rainbow trout were negatively affected by increasing dietary salt concentrations (Salman and Eddy 1988a). Furthermore, the influence of dietary salt on the growth of rainbow trout appeared to be size related, with larger fish tolerating higher dietary levels, but excessive salt levels deteriorated growth performance (Park et al. 1998). A significant decrease in growth performance was observed in juvenile silver catfish (*Rhamdia quelen*) fed diets with increasing salt contents up to 2% when maintained in neutral pH water (Copatti et al. 2011). Welker et al. (2012) found that no change in growth and feed efficiency in channel catfish, *Ictalurus punctatus*, occurred after feeding diets including salt up to 4% for 10 weeks. Likewise, Santos et al. (2014) fed cobia, *Rachycentron canadum*, with diets including gradually increasing salt up to 10% in low salinity water (5 g/L) for 40 days and found no significant change in growth performance but a deterioration in FCR with the increase of dietary salt. Therefore, our results in turbot maintained in brackish water are consistent with the latter research findings.

Dietary salt inclusion did not significantly change DFI but linearly reduced nitrogen, lipid, and energy intakes. Salt incorporation resulted in decrease in dietary protein, lipid, and energy levels (Table 1), which could partly play a role in reduced nitrogen and energy intakes. If the experimental approach to dietary formulation was based on iso-protein, iso-lipid, and iso-energetic principles, preferably on their digestible levels (Glencross 2020), the influence of salt levels on dietary nutrient intakes could be better understood. In fact, the dilution in nutrient levels due to the increase of dietary salt incorporation should have increased DFI and nitrogen intake, considering that protein- and energy-dense diets limit those in fish (Kaushik and Schrama 2022; Saravanan et al. 2012), but this did not occur in the present study. Therefore, the reductions in nitrogen, lipid, and energy intakes appear to be a result of the increase in dietary salt rather than the nutrient dilution, considering that ad libitum feeding was made to fish to regulate their feed consumption. The nutrient utilization variables, including nitrogen, and energy utilization, also showed a significant linear decrease in response to higher salt intakes. Therefore, the negative effects of dietary salt on turbot growth and nutrient utilization could be attributed to several factors, including increased energy expenditure due to osmoregulatory stress, reduced nutrient absorption, and increased excretion of water, when the findings of former studies are considered (Park et al. 1998; Salman 2009; Salman and Eddy 1987, 1988; Santos et al. 2014; Welker et al. 2012).

The whole-body dry matter and ash were quadratically affected by increasing dietary salt levels in this study. These results are partly consistent with those in rainbow trout, tilapia (*Oreochromis shiranus*), and rohu, which show decreased whole-body dry matter and a U-type parabola trend when fed a diet with increasing salt levels (Gangadhara et al. 2004; Mzengereza and Kang’ombe 2015; Park et al. 1998). Alam et al. (2015) found that the whole-body proximate composition of black seabass (*Centropristis striata*) was affected by increasing dietary salt inclusions up to 12.5% when they were reared in two different salinities (15‰ vs 10‰) with the lowest moisture and highest lipid levels in fish fed 5% salt diets in 10‰ salinity. An 8-month study in the same species but with larger individuals in low salinity revealed that a dietary increase in salt of up to 7.5% only affected the whole body protein by a linear decrease with the dietary salt levels (Alam et al. 2021). Salt supplementation to the diet of juvenile Chinook salmon reduced the whole body dry matter and lipid levels but did not change protein and ash levels after 6 weeks (Hanson et al. 2016). It is difficult to explain the reasons for the change in the whole body composition in the present study since the influence of dietary salt levels in fish appears to change depending on species, salinity, size, and feeding duration.

As a matter of fact, previous studies have reported the effects of dietary salt incorporation on osmoregulation via the gill and intestine with salinity (Con et al. 2017; Eroldoğan et al. 2005; Hallali et al. 2018; Salman 2009). For instance, parallel regulations in response to an increase in dietary salt additions and water salinities, including elevation of chloride cell proliferation, gill Na + and K + -ATPase activity in the gill (Salman and Eddy 1987), and changes in the localization of intestinal peptide transporters toward distal regions (Con et al. 2017; Hallali et al. 2018), have been reported. Therefore, a similar influence of salt addition on tissue fatty acid metabolism and composition can be expected to be influenced by water salinity (Kolosov and Kelly 2016). However, studies on salinity changes have reported controversial findings in terms of fatty acid metabolism in fish.

The salinity challenge of Atlantic salmon at 10 and 20‰ significantly reduced gill weight to body weight percentage and total lipid level of the gill (Takeuchi et al. 1989). Similarly, we observed a linear decrease in gill dry matter and protein concentrations in the present study. Takeuchi et al. (1989) observed a significant decrease in n-3 fatty acids in the gills of Atlantic salmon exposed to 10 and 20‰ salinities compared with freshwater. This finding is consistent with those in the present study, where an increase in dietary salt levels yielded linear reductions in 20:4n-6, 20:5n-3, and 22:6n-3. Similarly, an opposite relationship between salinity and ARA, EPA, and DHA concentrations in the gills of European sea bass and black seabream (*Acantahopagrus schlegelii*) has been reported by Cordier et al. (2002) and Li et al. (2022). The concentrations of 20:4n-6 and 22:6n-3 within the polar lipids of Atlantic salmon gill also similarly decreased after transition to seawater (Tocher et al. 2000). However, there appear to be species-specific differences in the gill fatty acid compositions of fish in response to salinity changes because sturgeon (*Acipencer naccarii*) and masu salmon (*O. masou*) showed increased 20:4n-6, 20:5n-3, and 22:6n-3 concentrations at higher salinity compared with freshwater (Li and Yamada 1992; Martínez-Álvarez et al. 2005). The change in Lc-PUFA levels in the gill tissue in the present study can be attributed to a factor other than beta-oxidation of the fatty acids since the substrate preference for the energetic use of fatty acids in the gill is limited regardless of salinity (Crockett et al. 1999). However, a dietary increase in salt can have elevated the use of Lc-PUFAs in a higher rate of epithelial cell proliferation in the gill tissue or the important roles of these fatty acids in osmoregulation due to osmotic stress (Evans and Kültz 2020; Glencross 2009; Laurent et al. 2006).

The liver is highly sensitive in terms of bioconversion of short-chain fatty acids to Lc-PUFAs and the concomitant fatty acid compositions to changes in water salinity in fish (Marrero et al. 2021; Morais et al. 2015; Sarker et al. 2011a). As such, the response pattern of fatty acid compositions of turbot liver to increasing dietary salt inclusions was significantly influenced by the changes in dietary salt levels in the present study, with higher SFA (mainly due to 16:0 and 18:0) and lower 18:3n-3 and EPA contents when fed diets with increasing salt levels. This finding is inconsistent with that reported by Li and Yamada (1992), who found elevated n-3 PUFA fatty acid levels in the liver of masu salmon exposed to seawater compared with freshwater. Our results are partly consistent with those reported by Sarker et al. (2011b), who recorded higher EPA concentrations in the liver of red sea bream (*Pagrus major*) maintained at 15 and 20‰ salinity than at 33‰. Moreover, some existing studies show that low-salinity waters enhance the bioconversion capacities of linoleic and α- linolenic acids into their LC-PUFAs, particularly ARA, EPA, and DHA compared with high-salinity waters in the livers of rabbitfish (*Siganus canaliculatus*) (Li et al. 2008) and Senegalese sole (*Solea senegalensis*) (Marrero et al. 2021). The decreases in 18:3n-3 and EPA levels with increasing dietary salt could be due to the increasing use of catabolism for energetic use.

PUFAs are the group of fatty acids that were most affected by dietary salt concentrations, represented by decreases in 18:3n-3 and 20:5n-3 but increases in 20:2n-6, 20:4n-6, and DHA in the filets. These results are partly in harmony with those reported by Yu et al. (2021), who found higher levels of 20:4n-6, EPA, and DHA in the flesh of red tilapia reared in increasing salinity. These researchers attributed this increase to the higher ability of fish to bioconvert 18:3n-3 and 18:2n-6 in brackish or saline water compared with freshwater. Similarly, gray mullet (*Chelon labrosus*) showed an increasing bioconversion of 18:3n-3 to n-3 LC-PUFAs in muscle when reared at 20‰ compared with those reared at 35‰ (Marrero et al. 2024). The results of the present study are inconsistent with those reported by Haliloǧlu et al. (2004), who found a higher level of 20:5n-3 but lower DHA in the filets of rainbow trout reared in freshwater than those kept in saline water. Further studies are needed to fully elucidate the influence of dietary salt or salinity on the fatty acid profile of turbot filets using molecular tools.

The results of this study demonstrate that increasing dietary salt levels have a significant impact on a number of key performance indicators of juvenile turbot reared in brackish water. While turbot can tolerate dietary salt levels of up to approximately 4%, higher salt levels lead to deterioration in growth and nutrient utilization performance. Nutrient utilization, including nitrogen, lipid, and energy intakes, significantly decreased with increasing salt levels, likely due to osmoregulatory stress and reduced nutrient absorption. Additionally, increasing dietary salt content altered the proximate composition and fatty acid profiles of various tissues. As fish meal includes a significant amount of salt and is increasingly being replaced with other protein sources (plant, insect, bacterial proteins, etc.) with lower salt levels, dietary inclusions of salt sources should be considered (Rimoldi et al. 2015). However, the influence of such applications on the fatty acid biosynthesis capacities and fatty acid profiles of turbot requires further exploration.

## Data Availability

No datasets were generated or analysed during the current study.
